# Beyond the Liver, Hepatitis E Can Affect the Nerves, Pancreas, and Blood Vessels. Extrahepatic Manifestations of Hepatitis E: A Comprehensive Literature Review

**DOI:** 10.7759/cureus.5499

**Published:** 2019-08-27

**Authors:** Veeraraghavan Meyyur Aravamudan, Shahab R Khan, Sushanth Dosala, Ikram Hussain

**Affiliations:** 1 Internal Medicine, Woodlands Health Campus, Singapore, SGP; 2 Internal Medicine, Banner University Medical Center, University of Arizona, Tucson, USA; 3 Internal Medicine, University of Illinois at Chicago, Illinois, USA; 4 Internal Medicine: Gastroenterology, Woodlands Health Campus, Singapore, SGP

**Keywords:** guillain-barré syndrome (gbs), extrahepatic manifestations, hepatitis e, pancreatitis, mixed cryoglobulinemia

## Abstract

Extrahepatic manifestations of Hepatitis E, though rare, are being increasingly reported in the medical literature. In this review article, we will discuss the extrahepatic manifestations of hepatitis E, such as Guillain-Barre syndrome, pancreatitis, and cryoglobulinemia, their clinical association with hepatitis E, and their management.

## Introduction and background

Hepatitis E virus (HEV) results primarily from human-to-human transmission through the fecal-oral route and is most prevalent in low-resource countries. It presents primarily as "acute viral hepatitis" syndrome, which is usually a self-limiting illness. A few cases progress to acute liver failure, a serious illness with a high mortality rate. Infection during pregnancy is associated with a higher risk of symptomatic disease, severe liver injury, and mortality. Severe disease has also been observed in persons with pre-existing chronic liver disease. Some cases have associated extrahepatic features, particularly acute pancreatitis, and neurological manifestations. Chronic infection appears to be extremely infrequent with these HEV genotypes [1].

## Review

Objectives

The objectives of this literature review is to discuss the extrahepatic manifestations of Hepatitis E and their complications and management plan.

Materials and methods

We conducted a literature search of journal articles using the US National Library of Medicine, PubMed, MEDLINE, Embase, Cochrane Library, and Google Scholar databases, ClinicalTrials.gov for studies, and ISI Web of Science. No date restrictions were placed on the search. A thorough search for controlled clinical trials and cohort studies was conducted. We used the keywords ”Extrahepatic manifestations” and “Hepatitis E."

Included studies were studies published in English that assessed the association between the extrahepatic manifestations of Hepatitis E. Reference lists were also screened. From the search results, articles with irrelevant titles were discounted, with the remaining abstracts examined for relevance.

The authors of this review independently determined the eligibility of studies and assessed the methodology of the included studies. In this review article, we will discuss the extrahepatic manifestations of Hepatitis E.

Extrahepatic manifestations of Hepatitis E

HEV infection appears to be strongly associated with acute pancreatitis, neurological disorders (with primarily dominant peripheral nerve involvement, most commonly manifested as Guillain-Barré syndrome, followed by neuralgic amyotrophy), hematological diseases (hemolytic anemia due to glucose phosphate dehydrogenase deficiency and severe thrombocytopenia), glomerulonephritis, and mixed cryoglobulinemia.

Table 1 outlines the findings of several studies on the extrahepatic manifestations of Hepatitis E.

**Table 1:** Review of studies done on the extrahepatic manifestations of Hepatitis E

Study Author(s)	Study Title	Findings
Bazerbachi et al. [2]	Extra-hepatic manifestations associated with hepatitis E virus infection: A comprehensive review of the literature	Fifty-three patients with acute pancreatitis (AP) associated with non-fulminant acute hepatitis E; further, 37 cases of Guillain-Barré Syndrome (GBS) were reported in 16 case reports and 2 case-controlled studies. There have only been 7 case reports and 3 small case series, with a total of 17 cases, published on severe hemolysis occurring in patients with acute hepatitis E associated with G6PD deficiency. Three documented cases of AIHA associated with HEV infection have been published, and 6 case reports and one case series including a total of 9 cases of thrombocytopenia. Finally, there were 11 patients associated with HEV causing mixed cryoglobulinemia.
Belbézier et al. [3]	Neurologic disorders and Hepatitis E: Review of literature	The author found 130 cases described between 2000 and 2017, the majority of which were associated with the genotype 3 and reported in Europe or in Asia. It affected immunocompetent (93%) men in particular, with a median age of 52 years. The main neurologic disorders were Guillain-Barré syndrome (54 cases), Parsonage-Turner syndrome (35 cases), multiplex mononeuropathy (6 cases), and meningitis and meningoencephalitis (9 cases).
Cheung et al. [4]	Review of the neurological manifestations of hepatitis E infection.	A literature review found 25 cases reporting neurological manifestations of HEV in both acute and chronic infection. Guillain-Barre syndrome and brachial neuritis are most frequently reported. Other reported disorders include transverse myelitis, cranial nerve palsies, seizure, and intracranial hypertension.
Dalton et al. [5]	Hepatitis E virus and neurological injury.	A literature review found 91 cases of HEV-associated neurological injury, most of which involve GBS, neuralgic amyotrophy, and encephalitis/myelitis.
Dalton et al. [6]	Hepatitis E virus infection and acute non-traumatic neurological injury: A prospective multicentre study.	Four hundred and sixty-four consecutive patients presenting to hospital with acute non-traumatic neurological illnesses were tested for HEV by serology and PCR from four centers in the UK, France, and the Netherlands. Eleven of these patients (2.4%) had evidence of current/recent HEV infection. Seven had HEV RNA identified in serum and four were diagnosed serologically. Neurological cases in which HEV infection was found included neuralgic amyotrophy (n=3, all PCR positive); cerebral ischemia or infarction (n=4); seizure (n=2); encephalitis (n=1); and an acute combined facial and vestibular neuropathy (n=1).
Fritz et al. [7]	Pathological cerebrospinal fluid findings in patients with neuralgic amyotrophy and acute Hepatitis E virus infection.	This study involved 35 patients with neuralgic amyotrophy and a control group for markers of HEV infection. Acute HEV infection was found in neuralgic amyotrophy (NA) patients only and was associated with an inflammatory response in the central nervous system. Shedding of HEV RNA into the cerebrospinal fluid and intrathecal production of anti-HEV immunoglobulin M occurred in one patient, suggesting that HEV is neurotropic.
Fukae et al. [8]	Guillain-Barré and Miller Fisher syndromes in patients with anti-hepatitis E virus antibody: a hospital-based survey in Japan	Sera obtained from 63 patients with GBS or Miller Fisher syndrome (MFS) and 60 control subjects were examined for both HEV-IgM and HEV-IgG. Of the 63 patients, 3 were positive for both HEV-IgM and elevated hepatic enzymes: 2 had GBS, and one had MFS. No control subjects were positive for HEV-IgM. Our study demonstrated that 4.8% of patients with GBS or MFS from our institution had associated acute HEV infection. There were no clinical differences between GBS with HEV infection and other GBS cases.
Geurtsvankessel et al. [9]	Hepatitis E and Guillain-Barre syndrome	A prospective case-control study was conducted between July 2006 and June 2007 enrolling 100 consecutive GBS cases from Dhaka Medical College Hospital, Bangabandhu Sheikh Mujib Medical University, and Dhaka Central Hospital in Dhaka, Bangladesh. Anti-HEV IgM seroprevalence was significantly higher among GBS patients. A case-control study among GBS patients in Bangladesh documented that 11/100 (11%) had an associated acute HEV infection. IgM seropositive individuals were tested for HEV RNA, yielding one positive serum sample classified as HEV GT1.
Haffar et al. [10]	Frequency and prognosis of acute pancreatitis associated with acute hepatitis E: A systematic review.	Thirteen case reports and 4 case series were found with 55 patients meeting the inclusion criteria. All patients originated from Southern Asia or had recently traveled to that area. The mean age at diagnosis was 28 years with a male to female ratio of 18:1. The mean interval between the onset of jaundice and the onset of AP pain was 10 days. AP was mild or moderately severe in 45 patients (82%) and severe in 10 patients (18%). Mortality was reported in 2 patients (3.6%).
Haffar et al. [11]	HEV-associated cryoglobulinemia and extrahepatic manifestations of hepatitis E.	Two previous reports described an association between mixed cryoglobulinemia and HEV infection.
Kamar et al. [12]	Hepatitis E virus and the kidney in solid-organ transplant patients.	The author reported 8 documented cases of mixed cryoglobulinemia in French patients who had undergone an organ transplant and had chronic hepatitis E, genotype 3. Three months after treatment with pegylated interferon or ribavirin, HEV clearance was achieved in all patients followed by the loss of cryoglobulinemia in all patients.
Kamar et al. [13]	Hepatitis E virus and neurologic disorders.	A recent case series from Southwest England and Toulouse found a 5.5% prevalence (7 out of 126 over five years) of neurological complications in locally acquired HEV infections (Hepatitis E virus and neurologic disorders). The study took place between 2004 and 2009 at 2 hospitals in the United Kingdom and France, among 126 patients with locally acquired acute and chronic HEV genotype 3 infection. Among these patients, neurologic complications developed in 7 (5.5%): inflammatory polyradiculopathy (n = 3), Guillain-Barre syndrome (n = 1), bilateral brachial neuritis (n = 1), encephalitis (n = 1), and ataxia/proximal myopathy (n = 1). Three cases occurred in non-immunocompromised patients with acute HEV infection, and 4 were in immunocompromised patients with chronic HEV infection.
Marson et al. [14]	Low prevalence of hepatitis E virus in type II mixed cryoglobulinemia.	Researchers screened 40 Italian patients with hepatitis C virus-related mixed cryoglobulinemia for anti-HEV antibodies and identified one patient with HEV co-infection. The patient had a history of jaundice 17 years before the detection of cryoglobulinemia, which could be presumptively attributed to a sporadic HEV infection. Since HEV RNA was not assessed, this case presents a probable HEV-related mixed cryoglobulinemia.
Mishra et al. [15]	Acute pancreatitis associated with viral hepatitis: a report of six cases with review of the literature.	The study found 6 reported cases of acute pancreatitis from Hepatitis E.
Pischke et al. [16]	HEV-associated cryoglobulinemia and extrahepatic manifestations of hepatitis E	This study reported a case of cryoglobulinemia associated with Hepatitis E.
Raj et al. [17]	Acute Hepatitis E-associated acute pancreatitis: A single-center experience and literature review.	Of 790 patients with AP, 16 (2.1) had hepatitis E and no other cause of AP; coexistent hepatitis A and B were present in two and one of them, respectively. Acute pancreatitis began a median of 8 days after acute hepatitis and was mild in 10 cases and severe in 6 cases. Complications included intra-abdominal collections (n=5), acute renal failure (n=4), and acute lung injury (n=2). Median bilirubin, alanine aminotransferase, and prothrombin time were 9.8 (0.4-25) mg/dL, 822 (54-4009) IU/L, 14.6 (9.7-27.4) seconds, respectively. Acute liver failure occurred in only one patient. No patient needed surgical, endoscopic, or percutaneous intervention.
Stevens et al. [18]	Diagnostic challenges and clinical characteristics of Hepatitis E virus-associated Guillain-Barré Syndrome.	The researchers determined the prevalence of HEV-associated GBS in a Belgian cohort, studied the clinical spectrum of HEV-associated GBS and discussed the difficulties in diagnosing acute HEV infection. A single-center, retrospective cohort study was conducted between January 1, 2007, and November 1, 2015. All patients with GBS or a GBS variant who presented to the adult neurology department of the University Hospital Leuven were identified via a search of the electronic medical records. Hepatitis E virus IgM and IgG reactivity was determined. In a subgroup, polymerase chain reaction for HEV was performed. Seventy-three eligible patients with GBS 6 (8%) showed positive reactivity on IgM assays for HEV, indicating a possible acute HEV infection. Thus, 4 patients (6%) in our cohort had probable acute HEV infection. Two of these patients presented with an infrequent GBS variant.
Van den Berg et al. [19]	Guillain-Barré syndrome associated with preceding hepatitis E virus infection.	In several case-controlled studies of GBS involving 201 patients in the Netherlands, acute HEV infection was associated with this syndrome in 5% of patients and only 0.5% of controls.
Van Ejik et al. [20]	Neuralgic amyotrophy and hepatitis E virus infection.	HEV testing was conducted in a retrospective cohort of 28 Cornish patients with NA (2011-2013) and a prospective cohort of 38 consecutive Dutch patients with NA (2004-2007). Five cases (10.6%) of acute hepatitis E infection were identified in a total group of 47 patients with NA of whom serum samples were available. Acute hepatitis E is found in 10% of patients with NA from the United Kingdom and the Netherlands.
Wang et al. [21]	Hepatitis E virus infection in acute non-traumatic neuropathy: A large prospective case-control study in China.	Determined the frequency and causal relationship of HEV in patients with non-traumatic neurological disorders in China, where GT4 HEV is prevalent. There were 1,117 consecutive patients diagnosed with neurological illnesses in a hospital of eastern China and 1,475 healthy controls who took the routine examination in the same hospital and were tested for HEV by serology and molecular methods. Anti-HEV IgM antibodies were detectable in 6 (0.54%) of the patients and 10 (0.68%) of the healthy controls (P = 0.651). Serum HEV RNA was detected in all 16 individuals with positive anti-HEV IgM. The 6 patients with HEV infection included 2 viral encephalitis, 2 posterior circulation ischemia, 1 peripheral neuropathy, and 1 GBS.
Woolson et al. [22]	Extra-hepatic manifestations of autochthonous hepatitis E infection.	Autochthonous, or locally acquired, hepatitis E is increasingly recognized in developed countries, and is thought to be a porcine zoonosis. The authors conducted a retrospective review of the data in 106 cases of autochthonous hepatitis E (105 acute and 1 chronic). Eight (7.5%) cases presented with neurological syndromes, which included brachial neuritis, GBS, peripheral neuropathy, neuromyopathy, and vestibular neuritis. One patient presented with a cardiac arrhythmia, 12 patients (11.3%) presented with thrombocytopenia, 14 (13.2%) with lymphocytosis, and 8 (7.5%) with a lymphopenia, none of which had any clinical consequence.

Results

Guillain-Barre Syndrome and HEV

It is recommended that clinicians consider the possibility of HEV infection in patients with neurological disorders and concurrent transaminase elevation, especially those with peripheral nerve involvement. The diagnosis may be suggested by HEV serology but should be confirmed with molecular testing in serum, cerebrospinal fluid (CSF), or both. The recognition of HEV infection in a patient presenting with neurological manifestations could present an opportunity to treat an active HEV infection with antivirals before chronic damage takes place, but further studies are needed to clarify their role in this setting [2].

The mechanisms of neurologic damage are unknown. Many viruses (including hepatotropic viruses) trigger neurologic signs and symptoms, especially Guillain-Barré syndrome. Such infections may elicit an immune response that cross-reacts with axolemmal or Schwann cell antigens and thereby damages peripheral nerves [13].

We recommend that clinicians strongly consider the possibility of HEV infection in patients with neurologic disorders, especially those with peripheral nerve involvement and liver abnormalities indicated by blood tests. The diagnosis may be suggested by HEV serology but should be confirmed by molecular documentation of HEV RNA in the serum, CSF, or both [13].

Neuralgic Amyotrophy and HEV

The mechanism by which HEV triggers neuralgic amyotrophy (NA) is uncertain. Direct infection of the brachial plexus cannot be excluded because HEV RNA was demonstrated in all HEV-associated patients at the start of their illness. Alternatively, HEV may trigger an immune response that damages the peripheral nervous system, similar to the pathogenesis of GBS.

At present, there is no evidence that HEV-related NA should be treated differently than any other forms of NA [20].

Acute Pancreatitis and HEV 

Acute pancreatitis associated with hepatitis E usually has a good prognosis. The mechanism of pancreatitis in patients with acute viral hepatitis (nonfulminant) is unknown, and it may be multifactorial. One proposed pathogenesis of pancreatitis associated with hepatitis is the development of edema of the ampulla of Vater with obstruction to the outflow of pancreatic fluid. A more plausible mechanism for virus-associated acute pancreatitis is the direct inflammation and destruction of pancreatic acinar cells by the virus [23].

In conclusion, acute pancreatitis is not uncommon. In a patient with acute viral hepatitis and acute or disproportionate abdominal pain, acute pancreatitis should be kept as a possibility. Conservative treatment leads to recovery in all the patients [23].

Cryoglobulinemia and HEV

Traditionally, Hepatitis C has been associated with cryoglobulinemia. In 2012, Kamar and colleagues reported eight documented cases of mixed cryoglobulinemia in French patients who had undergone an organ transplant and had chronic Hepatitis E, genotype 3. Three months after treatment with pegylated interferon or ribavirin, HEV clearance was achieved in all patients followed by the loss of cryoglobulinemia in all patients [12].

Pischke reported a case of HEV-associated cryoglobulinemia, no genotype documented, and symptoms resolved after HEV clearance [16]. Marson had earlier reported a probable case in 1995 of HEV cryoglobulenemia [14]. Del Bello reported a case of de novo membranoproliferative glomerulonephritis that occurred in a kidney transplant patient who developed a chronic HEV3 infection, which was successfully treated with ribavirin [24].

Guinault first documented a case of autochthonous HEV-induced cryoglobulinemic crescentic and membranoproliferative glomerulonephritis in an immunocompetent man with no notable medical history. He presented with edema, hypertension, increased serum creatinine level, and nephrotic syndrome. Type II cryoglobulinemia with monoclonal immunoglobulin G (IgG) κ light chain was detected. Anti-HEV IgG and IgM, as well as HEV RNA, were detected in serum and cryoprecipitate. Histologic analysis of a kidney biopsy specimen revealed features of crescentic and membranoproliferative glomerulonephritis. After HEV clearance, kidney and liver parameters improved and HEV RNA and cryoglobulinemia were undetectable [25].

There were only a few cases of Hepatitis E-associated cryoglobulenemia reported in the medical literature, all of these patients are chronic hepatitis patients, immunocompromised, all from western Europe, with genotype 3 confirmed in eight cases, with all MC type 2 or 3.

Antiviral treatment and immunosuppressive treatment are effective in all these cases and further clinical trials are needed to confirm these data.

Strengths of this literature review

The studies were done in multiple centers, which will increase the generalizability of the results within a population. The strengths of this review are in the systematic literature search with well-defined inclusion criteria, careful exclusion of redundant studies, the inclusion of good-quality studies with detailed extraction of data, and rigorous evaluation of study quality.

Limitations of this literature review

Most of the studies are retrospective and based on the small sample size and done in single centers. Therefore the risk of selection bias was unavoidable. Further, various aspects of interest are not included or not discussed in detail, such as race/ethnicity, medications, and outpatient care. The study is limited by the use of different practice patterns over the different centers and by the lack of a control group without infections. Most of the studies are done in a tertiary referral center and the intensity of liver disease from these centers may be more severe.

## Conclusions

Demyelinating polyneuropathies is known to be part of the complication from Hepatitis B and Hepatitis C infection. Again, it is worth screening for HEV in those patients with neuropathy and liver function abnormality. Physicians should be aware of the extrahepatic manifestations of Hepatitis E, as these manifestations have been reported in both acute and chronic cases. Physicians should also strongly consider the possibility of Hepatitis E infection in patients with neurologic disorders, especially in peripheral nerve and liver complications. The vast majority of cases of Hepatitis E are treated conservatively; however, a small number of patients can develop extrahepatic manifestations and physicians should look for these complications, as early diagnosis and treatment improve outcomes.
